# Supplementary material for: A Paratope-Enhanced Method to Determine Breadth and Depth TCR Clonal Metrics of the Private Human T-Cell Vaccine Response after SARS-CoV-2 Vaccination
Source: Int J Mol Sci. 2023 Sep 18;24(18):14223. doi: 10.3390/ijms241814223 (PMC10531868; doi:10.3390/ijms241814223)
Supplement: Supplementary file 1 [file ijms-24-14223-s001.zip › ijms-2531910-supplementary.pdf]

*Article*

# A Paratope-Enhanced Method to Determine Breadth and Depth TCR Clonal Metrics of the Private Human T-Cell Vaccine Response after SARS-CoV-2 Vaccination

**Dalin Li [1], Ana Jimena Pavlovitch-Bedzyk [2], Joseph E. Ebinger [3], Abdul Khan [1], Mohamed Hamideh [1], Akil Merchant [4], Jane C. Figueiredo [4], Susan Cheng [3], Mark M. Davis [2,5], Dermot P. B. McGovern [1], Gil Y. Melmed [1], Alexander M. Xu [4] and Jonathan Braun [1,*]**

## Supplementary materials

**Table S1.** Effect of anti-TNF immunotherapy on the T-cell response.

| comparison | outcome | N | estimate | se | stat | p |
| --- | --- | --- | --- | --- | --- | --- |
| | Breadth_Poisson | 224 | 0.048 | 0.039 | 1.225 | 0.222 |
| week2 vs dose1 | Depth_Poission | 224 | -0.048 | 0.040 | -1.206 | 0.229 |
| | Depth_gliph | 224 | -0.010 | 0.038 | -0.252 | 0.801 |
| | Breadth_gliph | 224 | 0.003 | 0.038 | 0.080 | 0.936 |
| | Breadth_Poisson | 251 | -0.062 | 0.038 | -1.646 | 0.101 |
| week8 vs dose1 | Depth_Poission | 251 | -0.042 | 0.038 | -1.110 | 0.268 |
| | Breadth_gliph | 251 | -0.028 | 0.039 | -0.719 | 0.473 |
| | Depth_gliph | 251 | 0.014 | 0.038 | 0.370 | 0.712 |
